# Targeting heparanase-associated epithelial glycocalyx remodeling attenuates Akt/NF-κB–related barrier dysfunction in experimental COPD

**DOI:** 10.3389/fphar.2026.1934045

**Published:** 2026-09-21

**Authors:** Yueping Bai, Xushan Ma, Hongzhen Jin, Junna Song, Xiaoxuan Zeng, Fengjuan Liu, He Fang, Xiao Wu, Song Yang, Wei Zhao

**Affiliations:** 1 State Key Laboratory of Medicinal Chemical Biology, College of Pharmacy, Key Laboratory of Molecular Drug Research and KLMDASR of Tianjin, Nankai University, Tianjin, China; 2 Qingdao Central Hospital, NHC Key Laboratory of Cardiopulmonary Rehabilitation and Functional Recovery, Industry-Academia-Research Collaborative and Innovation Center for Intelligent Rehabilitation Drug R&D of Shandong Province, Qingdao Key Laboratory of Precision Drug Research for Chronic Disease Rehabilitation, School of Health and Life Sciences, University of Health and Rehabilitation Sciences, Qingdao, China

**Keywords:** airway epithelial barrier, akt/NF-κB signaling, COPD, CV122, epithelial glycocalyx, heparanase inhibition

## Abstract

**Background:**

Chronic obstructive pulmonary disease (COPD) is characterized by persistent airway inflammation, epithelial barrier dysfunction, and progressive structural remodeling. Although glycocalyx impairment has been implicated in COPD pathogenesis, the mechanisms linking glycocalyx degradation to epithelial barrier injury remain incompletely understood. Heparanase (HPSE), the only mammalian endoglycosidase capable of degrading heparan sulfate, may contribute to COPD progression by regulating epithelial glycocalyx integrity.

**Methods:**

HPSE expression was evaluated in serum samples from patients with COPD, a murine COPD model induced by cigarette smoke exposure combined with lipopolysaccharide (LPS), and cigarette smoke extract (CSE)-stimulated human bronchial epithelial BEAS-2B cells. The therapeutic effects of CV122, a small-molecule heparanase inhibitor previously developed by our group, were investigated *in vivo* and *in vitro* using assessments of lung function, histopathology, inflammatory cytokine production, epithelial permeability, glycocalyx integrity, junctional protein expression, and Akt/NF-κB signaling activity.

**Results:**

HPSE abundance, including the mature 50-kDa HPSE form, was increased in serum from patients with COPD, lung tissues of COPD mice, and CSE-stimulated epithelial cells. In the murine COPD model, CV122 treatment significantly improved pulmonary function, reduced inflammatory cytokine production, and attenuated emphysematous and fibrotic remodeling. Mechanistically, CV122 preserved epithelial glycocalyx integrity, restored the expression and localization of ZO-1, occludin, and E-cadherin, and reduced epithelial and vascular permeability. In addition, CV122 inhibited Akt and NF-κB pathway activation, accompanied by decreased levels of TNF-α, IL-1β, and IL-6. Consistently, CV122 protected BEAS-2B cells against CSE-induced apoptosis, cytotoxicity, inflammatory responses, and barrier dysfunction.

**Conclusion:**

This study identifies HPSE-associated glycocalyx remodeling as an important contributor to epithelial barrier dysfunction in COPD. Pharmacological inhibition of HPSE with CV122 preserves glycocalyx integrity, restores epithelial barrier function, and attenuates inflammatory signaling, at least in part through suppression of the Akt/NF-κB pathway. These findings advance our understanding of glycocalyx remodeling in COPD and support HPSE as a promising therapeutic target for disease modification.

## 1 Introduction

Chronic obstructive pulmonary disease (COPD) is a progressive respiratory disorder characterized by persistent airflow limitation, chronic airway inflammation, and structural destruction of the lung parenchyma (Stolz et al., 2022; Christenson et al., 2022). Its pathogenesis is primarily driven by long-term exposure to noxious particles, particularly cigarette smoke, leading to airway remodeling and irreversible damage to lung tissue (Zhou et al., 2020; Yang et al., 2025; Celli et al., 2025). Clinically, COPD manifests as emphysema, small airway fibrosis, and progressive decline in lung function. The prevalence of COPD has continued to rise in recent years, with high morbidity and mortality, making it the fourth leading cause of chronic disease worldwide and posing a substantial burden on healthcare systems (Chen et al., 2023; Boers et al., 2023). Current standard therapies rely mainly on β_2_-adrenergic agonists and corticosteroids, which improve airway function and reduce inflammation, respectively (Wang et al., 2020). However, drug tolerance, adverse effects, and high relapse rates after discontinuation limit their long-term efficacy (Singh et al., 2025), highlighting the urgent need for novel therapeutic strategies with well-defined mechanisms and potential disease-modifying effects.

The airway epithelial barrier represents the first line of defense between the external environment and the lung parenchyma, and its structural integrity is essential for maintaining pulmonary homeostasis (Ji et al., 2026; Hiemstra et al., 2015; Varricchi et al., 2024). This barrier depends on multiple coordinated components: tight junctions and adherens junctions form a molecular “gate” controlling paracellular permeability, while the apical glycocalyx provides a glycosaminoglycan-rich biochemical shield (Otani, 2026; Li et al., 2021; Aghapour et al., 2018). Together, these structures regulate selective permeability, and disruption of this finely tuned system compromises the physical defense, exposes the underlying basement membrane, and triggers pathological cascades including innate immune activation, persistent airway inflammation, mucus hypersecretion, airway remodeling, and hyperresponsiveness, ultimately accelerating lung function decline and increasing the risk of acute exacerbations (Li et al., 2021; Brightling et al., 2025).

Among the glycocalyx components, heparan sulfate (HS) plays a central role by binding diverse cytokines, growth factors, and chemokines, thereby restricting their diffusion and maintaining local signaling microenvironments (Rana et al., 2026; Shaffer et al., 2024). Heparanase (HPSE) is the only known mammalian endo-β-D-glucuronidase capable of specifically degrading HS(17, 19). Its expression is markedly upregulated under inflammatory conditions (Changyaleket et al., 2017). HPSE-mediated HS degradation exerts dual pathological effects: it directly disrupts glycocalyx integrity, increasing pulmonary vascular permeability, and releases HS-bound proinflammatory mediators, amplifying inflammatory cascades and promoting tissue remodeling. (Rabelink et al., 2017; Farag et al., 2026; Vlodavsky et al., 2016). While HPSE has been extensively studied in cancer progression (Mo et al., 2025; Dai et al., 2017) and pulmonary, hepatic, and renal fibrosis (Cheng et al., 2022; Masola et al., 2015; Li et al., 2026), its precise role in COPD-associated airway epithelial barrier dysfunction, particularly regarding the temporal and molecular relationship between glycocalyx degradation and junctional protein disruption, remains poorly understood. In addition, research on the airway epithelial glycocalyx remains limited, hindering a comprehensive understanding of early barrier injury in COPD.

Building on previous evidence linking glycocalyx impairment and HPSE dysregulation to COPD-related lung injury, we investigated whether HPSE acts as an upstream enzymatic mediator of epithelial glycocalyx degradation and barrier dysfunction. HPSE expression was examined in human samples, while the effects of the small-molecule inhibitor CV122 were evaluated in CSE-stimulated epithelial cells and experimental COPD models. CV122, a previously reported small-molecule heparanase inhibitor developed by our group, has been shown to exhibit potent anti-HPSE activity in previous studies (Wang et al., 2024). CV122 alleviated structural and inflammatory injury and restored epithelial integrity. Mechanistically, CV122 preserved glycocalyx integrity and suppressed Akt/NF-κB signaling. These findings define an HPSE-centered glycocalyx–barrier–inflammation axis and support HPSE as a potential therapeutic target in COPD.

## 2 Methods

### 2.1 Human serum samples

This study was approved by the Ethics Review Committee of Qingdao Central Hospital, Rehabilitation University of China (approval number: KY202417101). Serum samples were collected from patients with confirmed COPD who were hospitalized in the Department of Respiratory and Critical Care Medicine. Non-COPD control subjects undergoing routine physical examinations were recruited concurrently as controls. All subjects provided written informed consent. Peripheral venous blood was collected from each subject and allowed to clot at room temperature for 30 min. After centrifugation at 3,000 rpm for 15 min, the supernatant serum was separated, aliquoted, and stored at −80 °C until further analysis. Demographic information available for the enrolled subjects is provided in Supplementary Table 1.

### 2.2 Animal

Male C57BL/6 J mice (6–8 weeks old) were obtained from Beijing Weitong Lihua Experimental Technology Co., Ltd. And housed under specific-pathogen-free conditions at the Nankai University Laboratory Animal Center with controlled temperature (25 °C) and humidity (40%–60%) and free access to food and water. All procedures were approved by the institutional animal ethics committee.

COPD was induced by combined intratracheal lipopolysaccharide (LPS) (Sigma, L2880) instillation and chronic cigarette smoke exposure. A total of 15 mice were randomly assigned to three groups (n = 5 per group): control, model, and CV122 treatment. LPS (20 μL, 1 mg/mL) was administered intratracheally on days 1 and 15, followed by brief rotation to ensure uniform lung distribution. Mice in the model and treatment groups were exposed to cigarette smoke for 1 h daily for 10 weeks, excluding instillation days. CV122 (20 mg/kg) was administered subcutaneously to the treatment group after each smoke exposure, while model mice received vehicle control. Control mice received saline and were maintained in a smoke-free environment. At the end of the study, mice were euthanized under deep anesthesia induced by inhaled isoflurane (RWD Life Science, R510-22) at 5% in oxygen until loss of reflexes and cessation of spontaneous breathing. Cervical dislocation was subsequently performed as a secondary physical method to confirm death. Lung tissues were immediately harvested for subsequent analyses.

### 2.3 Measurement of lung functions

To minimize pain and distress, mice were anesthetized with inhaled isoflurane using a precision vaporizer (3%–4% for induction and 1.5%–2% for maintenance in oxygen). Adequate anesthesia was confirmed by the absence of pedal withdrawal reflex before tracheal intubation. Pulmonary function was evaluated using the AniRes 2005 Animal Pulmonary Function Analysis System (Beijing Best Laboratory). Mice were anesthetized, intubated, and connected to the ventilatory system for measurement. Data acquisition was initiated after stabilization of the respiratory waveform. For each animal, measurements were performed in quintuplicate, and mean values were calculated. The assessed parameters included forced vital capacity (FVC), forced expiratory volume in 0.3 s (FEV0.3), and dynamic lung compliance (Cdyn).

### 2.4 Histological analysis

Lung tissues were sectioned at 4 μm thickness. Sections were stained with hematoxylin and eosin (H&E) (Solarbio, G1120) to evaluate cellular infiltration and structural changes, and with Masson’s trichrome (Solarbio, G1346) for collagen deposition. Mean linear intercept (MLI) was calculated using ImageJ. MLI was quantified according to previously described methods by measuring the mean distance between alveolar walls in randomly selected microscopic fields. For immunohistochemistry, sections underwent deparaffinization, heat-mediated antigen retrieval in citrate buffer, blocking, and incubation with primary antibodies (anti-HPSE (Proteintech, 24529-1-AP, 1:150), anti-ZO-1 (Proteintech, 21773-1-AP, 1:150), anti-E-cadherin (Proteintech, 20874-1-AP, 1:150), anti-occludin (Proteintech, 27260-1-AP, 1:150) overnight at 4 °C. After washing, sections were incubated with secondary antibodies for 2 h at room temperature. Antibody binding was visualized using DAB and counterstained with hematoxylin (Elabscience, E-IR-R213).

### 2.5 Micro-CT analysis

Mice were anesthetized with inhaled isoflurane using an isoflurane vaporizer (3%–4% for induction and 1.5%–2% for maintenance in oxygen) and positioned on the SkyScan 1276 CT scanner. Anesthesia was continuously maintained via a nasal cannula throughout the scanning procedure. Respiratory cycles were monitored using a respiratory gating system to minimize motion artifacts. High-resolution scanning was performed, and two-dimensional projection images were reconstructed into cross-sectional images. A three-dimensional digital voxel model of the thoracic cavity was generated using the accompanying software.

### 2.6 Evans blue staining

Evans Blue (0.5% in sterile saline) was filtered (0.22 μm) and stored at 4 °C in the dark. Mice received tail vein injections and were allowed 1 h for circulation. After euthanasia as described above, the thoracic cavity was opened, and hearts were perfused with chilled saline until the effluent became clear. Lungs were excised, weighed, incubated in formamide (1 mL per 100 mg tissue) at 37 °C for 48 h in the dark, centrifuged at 12,000 rpm for 30 min, and absorbance of the supernatant was measured at 630 nm to quantify Evans Blue content (Solarbio, E8010).

### 2.7 Giemsa staining

Separate the supernatant from the cells by centrifuging the bronchoalveolar lavage fluid. Spread the cells onto a microscope slide using a cell spreader and fix them with methanol. Next, stain the fixed cells with Giemsa stain, rinse them under running water, and examine them under a microscope for morphological identification (Solarbio, G1015).

### 2.8 ELISA assay

Concentrations of TNF-α (BioLegend, 430940), IL-1β (BioLegend, 432604), and IL-6 (BioLegend, 431304) in cell culture supernatants, mouse serum, and bronchoalveolar lavage fluid (BALF), HPSE levels in human serum (Shanghai Jianglai Biotechnology, JL11914) and mouse serum (Shanghai Jianglai Biotechnology, JL52918), HS levels in mouse serum (Nanjing Boyan Biotechnology, BY-EM221695), and HS levels in cell culture supernatants (Shanghai Jianglai Biotechnology, JL19659) were measured using commercially available ELISA kits according to the manufacturers’ instructions.

### 2.9 Cell culture and model establishment

Human bronchial epithelial BEAS-2B cells were cultured in Dulbecco’s modified Eagle’s medium (DMEM) supplemented with 10% fetal bovine serum (FBS) and 1% penicillin–streptomycin at 37 °C in a humidified atmosphere containing 5% CO_2_. Cigarette smoke extract (CSE) was freshly prepared by bubbling smoke from 10 commercial cigarettes into 5 mL of serum-free DMEM. Standardization was performed by measuring the absorbance of a 100 μL aliquot, and preparations with an absorbance value of 1.0 at 450 nm were designated as 100% stock solution. The stock was then filtered through a 0.22 μm membrane and diluted with culture medium to the desired concentrations immediately before use. Cells were exposed to CSE at the indicated concentrations and durations according to the experimental design.

### 2.10 Cell viability and LDH assay

For the concentration screening assay, BEAS-2B cells were seeded into 96-well plates at a density of 5 × 10^3^ cells per well. After 24 h of culture to allow adherence, the medium was replaced with fresh medium containing various concentrations of CSE, and cells were incubated for an additional 12 h. Subsequently, 20 μL of MTT (Solarbio, M8180) solution (5 mg/mL) was added to each well, followed by incubation at 37 °C under 5% CO_2_ for 4 h. The supernatant was aspirated, and 150 μL of DMSO was added to each well to dissolve the formazan crystals. After thorough mixing by oscillation, the absorbance at 490 nm was measured using a microplate reader to calculate cell viability.

For the drug treatment experiment, the cells were inoculated and adhered as described above. Before the experiment, the cells were starved for 2 h, then stimulated with 0.9% CSE for 12 h, and subsequently incubated with different concentrations of CV122 (25, 50 and 100 μM) for 24 h. Cell viability was assessed using the aforementioned MTT method. Additionally, cell culture supernatants and mouse BALF were collected, and lactate dehydrogenase (LDH) release levels were quantified according to the LDH release assay kit (Nanjing Jiancheng Bioengineering Institute, A020-2-2) instructions by measuring absorbance at 440 nm.

### 2.11 Apoptosis assay

Cells were seeded, treated, and collected as described above, then stained with Annexin V/propidium iodide according to the manufacturer’s instructions, and analyzed by flow cytometry within 1 h. At least 10,000 events were acquired per sample, and the percentage of apoptotic cells was quantified (Elabscience, E-CK-A211).

### 2.12 FITC-dextran assay

Adherent cells were seeded onto Transwell inserts to form monolayers. After treatment, cells were washed and incubated with 1 mg/mL FITC-dextran (Merck Millipore, 60,842-46–8) in the upper chamber for 2 h. Fluorescence in the lower chamber was measured (excitation 490 nm, emission 520 nm) to assess barrier permeability.

### 2.13 Western blot

Proteins were extracted from lung tissues or cells using RIPA buffer containing protease and phosphatase inhibitors. Protein concentration was determined by BCA assay (Solarbio, PC0020). Samples were separated by 10% SDS-PAGE, transferred to PVDF membranes (Merck Millipore, ISEQ00010), and incubated with primary antibodies. For HPSE analysis, anti-HPSE antibody (Abcam, ab288438, 1:1,000) was used, and the mature 50-kDa HPSE band was quantified as the enzymatically active form of HPSE. The remaining primary antibodies included anti-β-tubulin (Proteintech, 66240-1-Ig, 1:2,500), anti-AKT (affinity, AF0836, 1:1,000), anti-Phospho-AKT (Ser473) (affinity, AF0016, 1:1,000), anti-NF-κB p65 (Proteintech, 10745-1-AP, 1:1,000), anti-Phospho-NF-κB p65 (Ser468) (Proteintech, 82335-1-RR, 1:1,000), anti-IκB-α (Proteintech, 10268-1-AP, 1:1,000), anti-Phospho-IκB-α (Ser32/36) (Proteintech, 82349-1-RR, 1:1,000), anti-ZO-1 (Proteintech, 21773-1-AP, 1:1,000), anti-E-cadherin (Proteintech, 20874-1-AP, 1:1,000), anti-occludin (Proteintech, 27260-1-AP, 1:1,000) overnight at 4 °C, followed by secondary antibody incubation. Signals were visualized using enhanced chemiluminescence and quantified with ImageJ.

### 2.14 Immunofluorescence

Frozen lung sections (7 μm) were incubated with primary antibodies (anti-HPSE, anti-ZO-1, anti-E-cadherin, anti-occludin) overnight at 4 °C, followed by fluorescent secondary antibodies for 1 h. Cells were fixed, blocked, incubated with antibodies, and counterstained with DAPI. For wheat germ agglutinin (WGA) staining, samples were incubated with WGA (Gene Tex, GTX01502) at room temperature for 30 min, followed by thorough washing. For mitochondrial staining, cells were incubated with Mito-Tracker Green (Beyotime Biotechnology, C1048) at a recommended concentration for 30 min at 37 °C, then washed. Imaging was performed with a Zeiss LSM800 confocal microscope, and fluorescence intensity was quantified using ImageJ.

### 2.15 Statistical analysis

All histological and image-based quantitative analyses were performed by investigators blinded to the experimental group assignments. Data are presented as mean ± SD. Statistical analyses were performed using GraphPad Prism 8.0. Comparisons between two groups were performed using an unpaired two-tailed Student’s t-test. Comparisons among multiple groups were performed using one-way ANOVA followed by Tukey’s multiple-comparisons test. A value of P < 0.05 was considered statistically significant. No data points were excluded, and no formal outlier analysis was performed.

## 3 Results

### 3.1 HPSE is upregulated in clinical and experimental models of COPD

Previous studies have implicated glycocalyx disruption in the pathogenesis of COPD; however, the contribution of HPSE, a key enzyme mediating HS degradation, remains poorly defined. We therefore first assessed HPSE expression in serum samples from COPD patients and healthy controls. ELISA analysis showed increased serum HPSE levels in COPD patients (Figure 1A), suggesting an association between HPSE dysregulation and COPD. To further examine this relationship in experimental settings, we established a murine COPD model using combined intratracheal LPS instillation and chronic cigarette smoke exposure (Figure 1B). Histological analyses showed progressive pathological changes in the lungs of COPD mice. H&E staining revealed airway remodeling characterized by inflammatory cell infiltration and disruption of alveolar architecture at 30 days, with more severe alterations observed at 60 days. Masson staining further demonstrated a time-dependent increase in collagen deposition in peribronchial and interstitial regions. Quantitative analyses showed significant increases in mean linear intercept and fibrotic area in COPD mice, particularly at 60 days, confirming the successful establishment of the COPD model (Supplementary Figure S1A; Supplementary Figure S1B).

**FIGURE 1:**
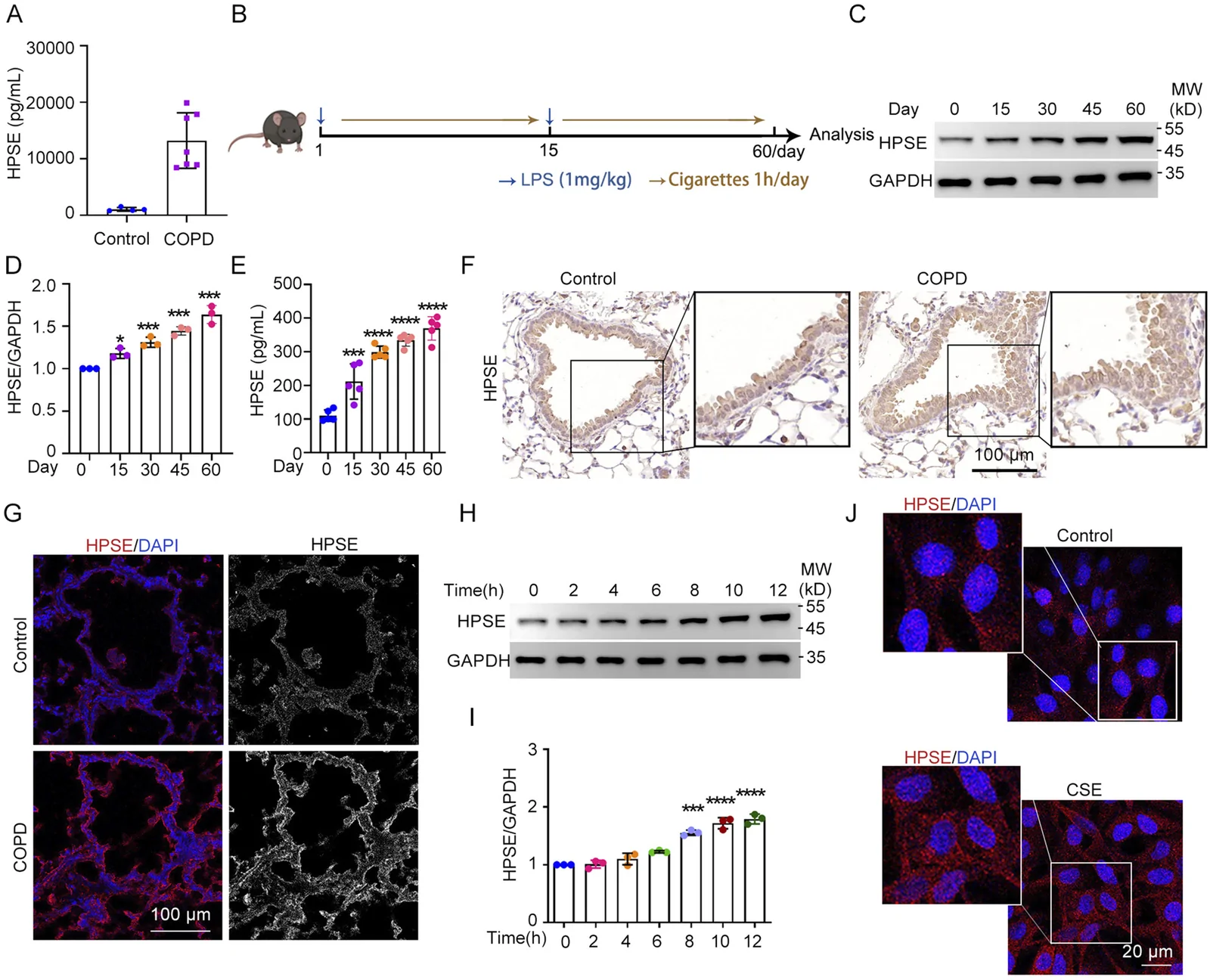
HPSE expression is elevated in human COPD samples and experimental models. **(A)** Serum HPSE levels in healthy controls (n = 4) and COPD patients (n = 7) measured by ELISA. **(B)** Schematic illustration of the experimental design for COPD induction in mice. **(C–D)** Representative Western blots and quantitative analysis of the mature 50-kDa HPSE form in mouse lung tissues during COPD progression. **(E)** Serum HPSE levels in mice at the indicated time points measured by ELISA. **(F)** Representative immunohistochemical staining of HPSE in lung tissues from control and COPD mice. **(G)** Representative immunofluorescence images showing HPSE expression in mouse lungs. **(H–I)** Representative Western blots and quantitative analysis of the mature 50-kDa HPSE form in BEAS-2B cells following CSE stimulation. **(J)** Representative immunofluorescence images of HPSE in control and CSE-treated BEAS-2B cells. Data are presented as mean ± SD. Mouse experiments: n = 5 mice per group; cell experiments: n = 3 independent experiments. *P < 0.05, **P < 0.01, ***P < 0.001, ****P < 0.0001; ns, not significant.

Following validation of the COPD model, we next examined HPSE in lung tissues. Western blot analysis demonstrated a significant increase in the mature 50-kDa HPSE form in COPD mice, while ELISA revealed elevated HPSE levels in serum (Figures 1C–E). At day 60, immunohistochemical and immunofluorescence analyses further showed increased HPSE expression predominantly localized to airway epithelial cells and adjacent interstitial regions (Figures 1F,G; Supplementary Figure S1C; Supplementary Figure S1D).

To establish an *in vitro* model of cigarette smoke–induced epithelial injury, BEAS-2B cells were exposed to increasing concentrations of CSE for 12 h. MTT analysis showed a dose-dependent reduction in cell viability, with significant cytotoxicity observed at concentrations ≥1.1% (Supplementary Figure S1E). Accordingly, 0.9% CSE was selected for subsequent experiments, as it induced moderate epithelial injury while preserving sufficient cell viability for mechanistic studies. Using this model, we next examined HPSE expression in response to cigarette smoke exposure. CSE stimulation induced time- and dose-dependent increases in the mature 50-kDa HPSE form, accompanied by increased intracellular HPSE immunofluorescence intensity (Figures 1H–J; Supplementary Figure S1F **)**.

Together, these findings suggest that HPSE is upregulated during cigarette smoke–induced epithelial injury and may contribute to COPD-related airway remodeling.

### 3.2 CV122 attenuates COPD progression and lung injury *in vivo* and *in vitro*

To evaluate the protective effects of CV122, a murine COPD model was established by combined intratracheal LPS instillation and chronic cigarette smoke exposure. CV122, a novel HPSE inhibitor, was administered subcutaneously throughout the disease induction period starting from day 1 of modeling, with control and COPD model groups included for comparison (Figure 2A). The effects of CV122 on COPD progression were systematically evaluated using pulmonary function testing, inflammatory profiling in BALF, histopathological assessment, and micro-CT imaging.

**FIGURE 2:**
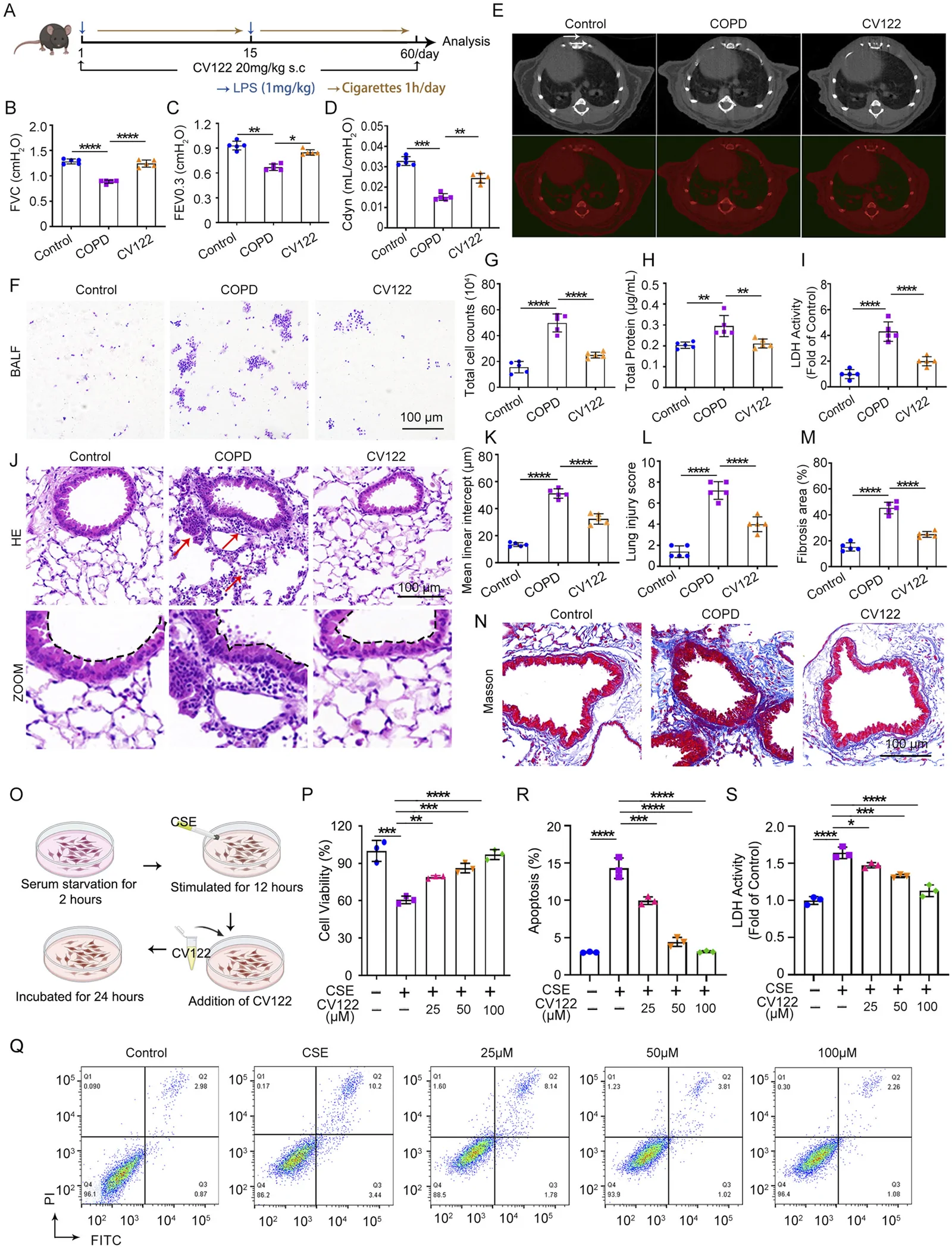
CV122 ameliorates COPD-associated pulmonary dysfunction and structural remodeling *in vivo* and *in vitro*. **(A)** Experimental design for COPD induction and CV122 treatment in mice. **(B–D)** Pulmonary function parameters, including FVC, FEV0.3, and Cdyn. **(E)** Representative micro-CT images of mouse lungs. **(F–G)** Representative Giemsa staining and quantification of inflammatory cells in BALF. **(H–I)** Total protein concentration and LDH activity in BALF. **(J–L)** Representative H&E staining and quantitative analysis of mean linear intercept (MLI) and lung injury scores. **(M–N)** Representative Masson’s trichrome staining and quantification of collagen deposition. **(O)** Schematic illustration of *in vitro* experimental design for CV122 treatment. **(P–R)** Cell viability and apoptosis analysis in BEAS-2B cells exposed to CSE with or without CV122 treatment. **(S)** LDH release in culture medium. Data are presented as mean ± SD. Mouse experiments: n = 5 mice per group; cell experiments: n = 3 independent experiments. *P < 0.05, **P < 0.01, ***P < 0.001, ****P < 0.0001; ns, not significant.

Pulmonary function analysis demonstrated significant reductions in FVC, FEV0.3, and Cdyn in COPD mice, whereas CV122 treatment markedly improved these parameters, indicating preserved lung function and improved respiratory mechanics (Figures 2B–D). Consistently, micro-CT imaging of COPD mice demonstrated prominent linear opacities and increased lung density, indicative of airway inflammation, alveolar destruction, and interstitial remodeling. These abnormalities were substantially reduced following CV122 treatment (Figure 2E). In BALF analysis, CV122 significantly reduced inflammatory cell infiltration (Figures 2F,G; Supplementary Figure S2), decreased total protein concentration (Figure 2H), and normalized LDH activity (Figure 2I), suggesting attenuation of pulmonary injury and reduced alveolar leakage. Histopathological examination further confirmed these findings. H&E staining revealed severe alveolar destruction, epithelial disruption, and inflammatory cell infiltration in COPD mice, whereas these pathological alterations were markedly alleviated by CV122 (Figures 2J–L). In addition, Masson’s trichrome staining demonstrated excessive perivascular and interstitial collagen deposition in COPD lungs, which was significantly reduced following CV122 treatment, indicating suppression of fibrotic remodeling (Figures 2M,N). *In vitro*, BEAS-2B cells were exposed to CSE and treated with increasing concentrations of CV122 (Figure 2O). CV122 dose-dependently restored cell viability (Figure 2P). Consistently, Annexin V-FITC/PI flow cytometric analysis demonstrated that CSE significantly induced epithelial apoptosis, whereas CV122 treatment, particularly at 50 μM, markedly reduced apoptotic cell populations (Figures 2Q,R). Furthermore, CV122 significantly decreased LDH release (Figure 2S), indicating improved membrane integrity and reduced cytotoxic injury.

Collectively, these findings demonstrate that CV122 exerts robust protective effects against COPD progression by attenuating inflammation, preserving epithelial integrity, reducing apoptosis, and suppressing structural and fibrotic lung remodeling both *in vivo* and *in vitro*. To further evaluate the safety of prolonged CV122 administration under physiological conditions, healthy mice receiving CV122 were examined. No significant differences were observed in pulmonary function, lung histology, or WGA staining compared with untreated healthy controls (Supplementary Figure S3), suggesting that CV122 does not measurably affect basal pulmonary physiology or glycocalyx organization under normal conditions.

### 3.3 CV122 preserves alveolar epithelial glycocalyx integrity in association with HPSE suppression

To determine whether CV122 protects the pulmonary epithelial barrier via HPSE inhibition, we comprehensively assessed HPSE expression, glycocalyx integrity, and pulmonary barrier function in both *in vivo* and *in vitro* models. Immunofluorescence and immunohistochemical analyses revealed markedly increased HPSE expression in the lungs of COPD mice, predominantly localized to airway epithelial and peribronchial regions. Notably, CV122 treatment significantly attenuated this upregulation (Figures 3A,B; Supplementary Figure S4A; Supplementary Figure S4B). Consistently, Western blot analysis further demonstrated a marked increase in the mature 50-kDa HPSE form in COPD lungs, which was significantly reduced following CV122 administration (Figures 3C,D). In addition, HPSE concentrations in both serum and BALF were significantly elevated in COPD mice and were significantly reduced following CV122 treatment (Figure 3E; Supplementary Figure S4C). Given the critical role of HPSE in degrading HS and disrupting the epithelial glycocalyx, we next evaluated glycocalyx integrity. WGA staining demonstrated a pronounced loss of glycocalyx coverage in COPD lungs, whereas CV122 treatment largely restored WGA fluorescence intensity, indicating preservation of epithelial surface glycocalyx structure (Figures 3F,G). To further determine whether HPSE-associated glycocalyx remodeling involved its specific substrate, HS levels were first assessed in mouse serum by ELISA. Compared with the control group, serum HS levels were significantly altered in COPD mice and were partially restored following CV122 treatment (Figure 3H). Consistently, immunofluorescence staining of mouse lung tissues showed markedly reduced HS fluorescence in COPD mice, whereas CV122 treatment significantly preserved HS abundance (Figures 3I,J). These complementary findings provide HS-specific evidence of glycocalyx remodeling and support an association between HPSE inhibition and preservation of HS integrity *in vivo*. Functionally, Evans blue extravasation assays showed significantly increased vascular and alveolar barrier permeability in COPD mice, which was markedly reduced by CV122, suggesting restoration of pulmonary barrier integrity (Figures 3K,L).

**FIGURE 3:**
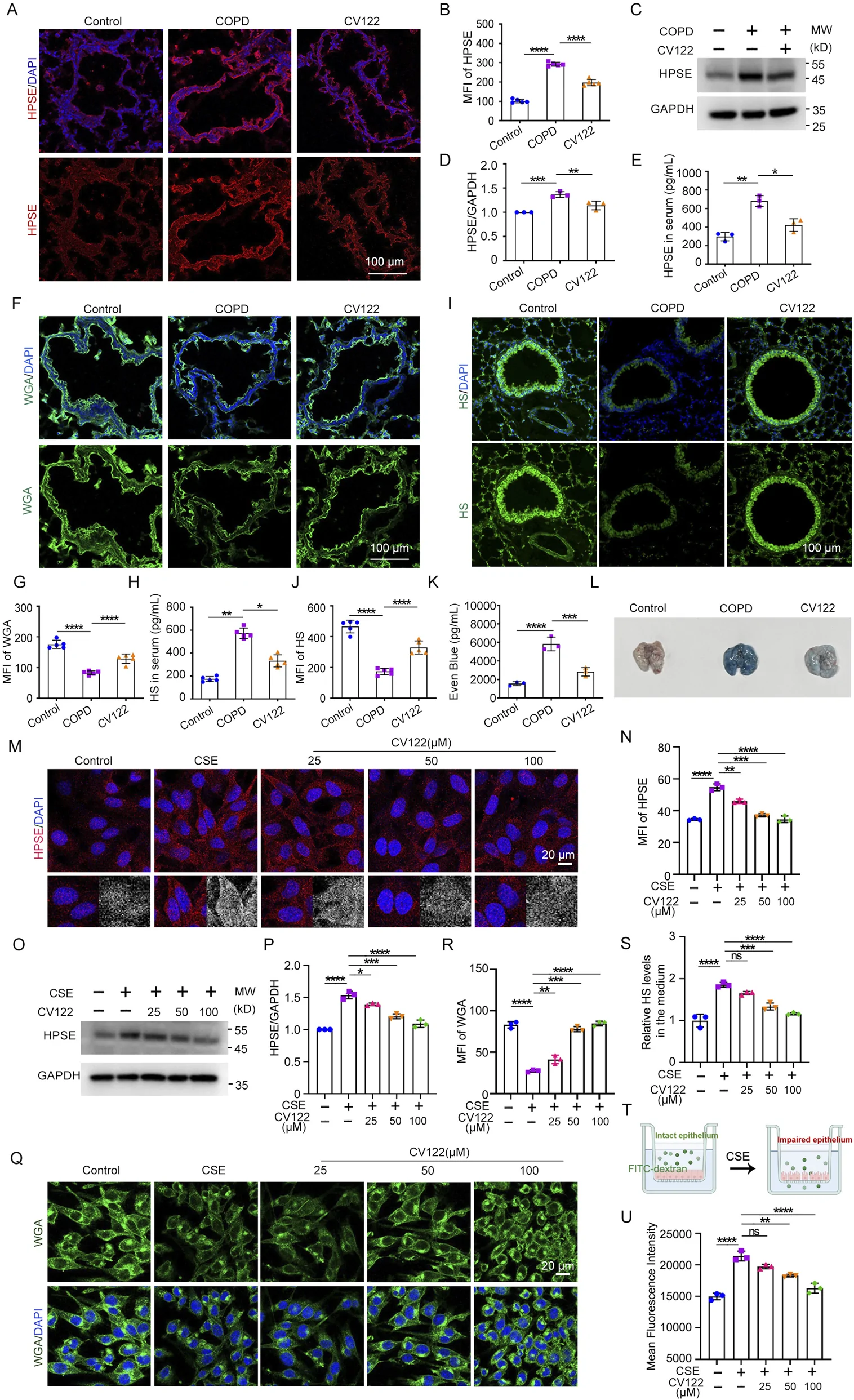
CV122 attenuates HPSE upregulation, preserves glycocalyx integrity, and restores epithelial barrier function in COPD models. **(A–B)** Representative immunofluorescence images and quantification of HPSE expression in lung tissues of control, COPD, and CV122-treated mice. **(C–D)** Representative Western blots and densitometric analysis of the mature 50-kDa HPSE form in lung tissues. **(E)** HPSE concentrations in serum measured by ELISA. **(F–G)** Representative WGA staining and quantitative analysis showing glycocalyx integrity in lung tissues. **(H)** ELISA analysis of HS levels in mouse serum. **(I)** Representative immunofluorescence images showing HS (green) expression in lung tissues from Control, COPD, and CV122-treated mice. Nuclei were counterstained with DAPI (blue). **(J)** Quantification of HS fluorescence intensity in lung tissues. **(K–L)** Representative Evans blue extravasation images and quantification of pulmonary vascular permeability. **(M–N)** Representative immunofluorescence images and quantification of HPSE expression in BEAS-2B cells exposed to CSE with or without CV122 treatment. **(O–P)** Representative Western blots and densitometric analysis of the mature 50-kDa HPSE form in BEAS-2B cells. **(Q–R)** Representative WGA staining and quantification of epithelial glycocalyx integrity in BEAS-2B cells. **(S)** ELISA analysis of HS levels in the culture supernatants of BEAS-2B cells treated with CSE in the absence or presence of the indicated concentrations of CV122. **(T)** Schematic illustration of CSE-induced epithelial glycocalyx disruption and barrier dysfunction. **(U)** Quantification of FITC-dextran permeability assay reflecting epithelial barrier function. Data are presented as mean ± SD. Mouse experiments: n = 5 mice per group; cell experiments: n = 3 independent experiments. *P < 0.05, **P < 0.01, ***P < 0.001, ****P < 0.0001; ns, not significant.

To further validate these findings *in vitro*, BEAS-2B cells were exposed to CSE to model epithelial injury. Immunofluorescence analysis showed that CSE markedly increased cellular HPSE expression, whereas Western blot analysis demonstrated a corresponding increase in the mature 50-kDa HPSE form. Both changes were dose-dependently attenuated by CV122 treatment (Figures 3M–P). Correspondingly, WGA staining revealed that CSE disrupted epithelial glycocalyx integrity, while CV122 preserved surface glycocalyx structure in a concentration-dependent manner (Figures 3Q,R). Consistent with the *in vivo* findings, ELISA analysis of BEAS-2B cell culture supernatants demonstrated significant alterations in HS levels following CSE stimulation, which were markedly reversed by CV122 treatment (Figure 3S). Functionally, CSE markedly increased FITC-dextran permeability across epithelial monolayers, indicating impaired barrier function; this effect was significantly attenuated by CV122 in a dose-dependent manner (Figures 3T,U), supporting restoration of epithelial barrier integrity.

Collectively, these data demonstrate that CV122 preserves pulmonary epithelial barrier function in both COPD mice and CSE-induced epithelial injury models, accompanied by reduced HPSE expression and mature active HPSE abundance, preservation of glycocalyx integrity, and decreased barrier permeability.

### 3.4 CV122 inhibits akt/NF-κb signaling and maintains epithelial barrier integrity *in vivo* and *in vitro*

Inflammatory responses were first evaluated by ELISA. Levels of IL-1β, IL-6, and TNF-α were significantly elevated in both BALF and serum from COPD mice, whereas CV122 treatment markedly reduced these pro-inflammatory cytokines, indicating effective suppression of systemic and pulmonary inflammation (Figures 4A–F). We next investigated whether the barrier-protective effects of CV122 were associated with modulation of inflammatory signaling pathways. Western blot analysis demonstrated that COPD induction significantly increased phosphorylation of Akt, IκBα, and NF-κB p65, while total protein levels of Akt, IκBα, and NF-κB p65 remained largely unchanged. Importantly, CV122 treatment markedly attenuated these phosphorylation events, suggesting inhibition of Akt/NF-κB pathway activation rather than changes in total protein abundance (Figures 4G–J). Consistently, immunofluorescence staining revealed that COPD disrupted the expression and membrane localization of key epithelial tight junction proteins, including ZO-1, Occludin, and E-cadherin, reflecting impaired epithelial barrier integrity. In contrast, CV122 treatment restored both protein expression and continuous junctional distribution along the epithelial layer, indicating structural recovery of the airway epithelial barrier (Figure 4K; Supplementary Figure S5).

**FIGURE 4:**
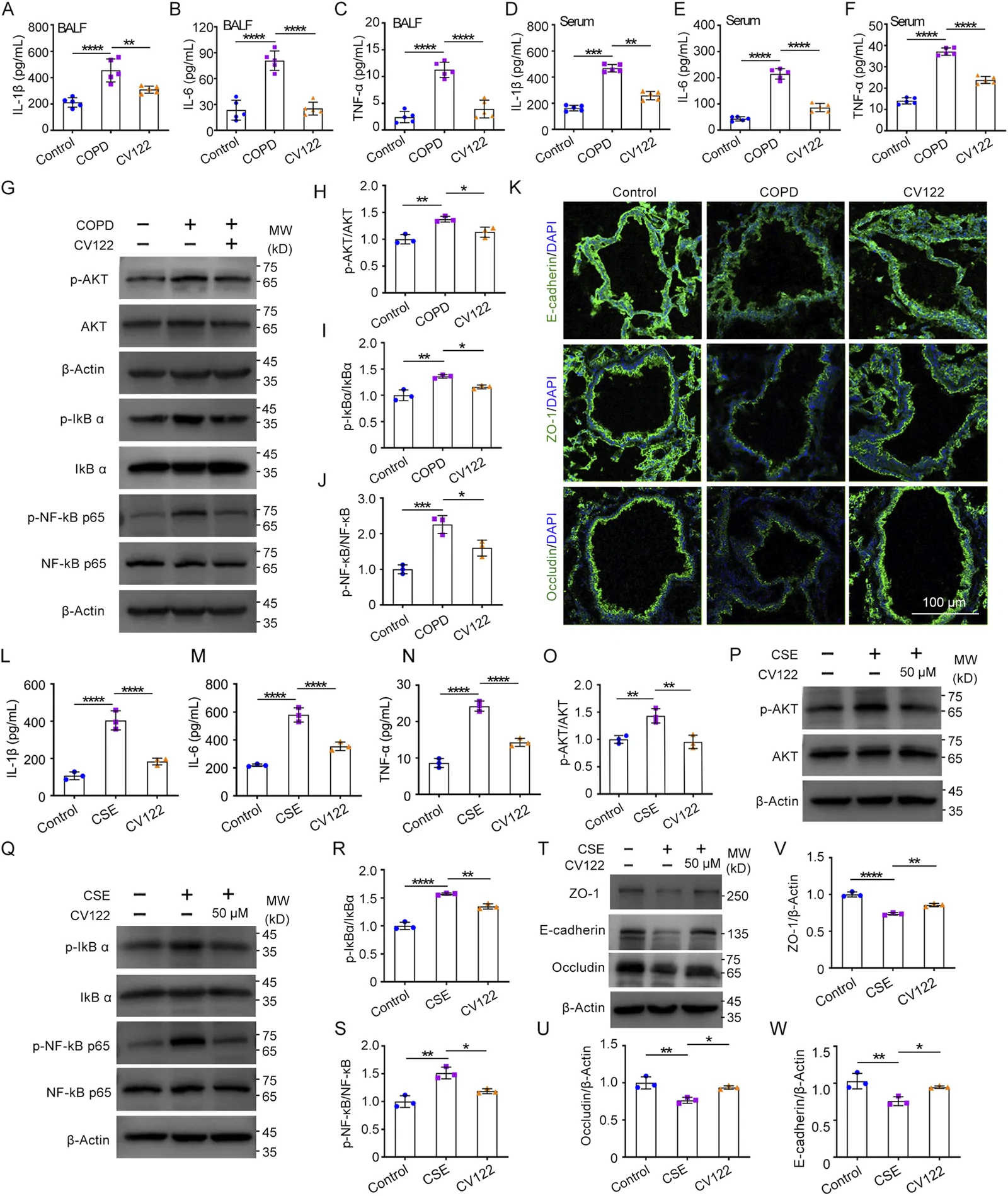
CV122 suppresses inflammation and restores epithelial barrier via Akt/NF-κB inhibition *in vivo* and *in vitro*. **(A–C)** Levels of IL-1β, IL-6, and TNF-α in BALF measured by ELISA. **(D–F)** Serum levels of IL-1β, IL-6, and TNF-α measured by ELISA. **(G–J)** Representative Western blots and densitometric analysis of Akt/NF-κB signaling pathway activation in lung tissues, including p-AKT/AKT, p-IκBα/IκBα, and p-NF-κB p65/NF-κB p65. **(K)** Representative immunofluorescence images showing expression of epithelial junction proteins, including E-cadherin, ZO-1, and Occludin, in lung tissues. **(L–N)** Levels of IL-1β, IL-6, and TNF-α in cell culture supernatants from BEAS-2B cells exposed to CSE with or without CV122 treatment. **(O–S)** Representative Western blots and densitometric analysis of Akt/NF-κB pathway activation in BEAS-2B cells, including p-AKT/AKT, p-IκBα/IκBα, and p-NF-κB p65/NF-κB p65. **(T–W)** Representative Western blots and quantitative analysis of epithelial tight junction proteins (ZO-1, Occludin, and E-cadherin) in BEAS-2B cells. Data are presented as mean ± SD. Mouse experiments: n = 5 mice per group. Cell experiments: n = 3 independent experiments, as indicated. *P < 0.05, **P < 0.01, ***P < 0.001, ****P < 0.0001; ns, not significant.

We further examined whether CV122 also modulated the inflammatory response associated with epithelial injury *in vitro*. ELISA analysis showed that CSE markedly increased the secretion of TNF-α, IL-6, and IL-1β in BEAS-2B cells, all of which were dose-dependently reduced by CV122 treatment (Figures 4L–N). We further validated these findings *in vitro* using a CSE-induced BEAS-2B cell model. Western blot analysis showed that CSE significantly activated the Akt/NF-κB signaling pathway, as evidenced by increased phosphorylation of Akt and NF-κB p65, whereas CV122 markedly suppressed these phosphorylation events (Figures 4O–S). In parallel, CSE reduced the expression of tight junction proteins (ZO-1, Occludin, and E-cadherin), while CV122 treatment restored their expression in a protective manner, further supporting its role in maintaining epithelial junctional integrity (Figures 4T–W).

Collectively, these results demonstrate that CV122 protects airway epithelial barrier function in both COPD mice and CSE-induced epithelial injury models by suppressing inflammatory cytokine production, inhibiting Akt/NF-κB signaling activation, and preserving epithelial tight junction integrity.

## 4 Discussion

Our previous studies established CV122 as a selective HPSE inhibitor and demonstrated its protective effects in experimental sepsis (Wang et al., 2024). More recently, we showed that HPSE inhibition attenuates glycocalyx degradation, epithelial–mesenchymal transition, and pulmonary fibrosis (Li et al., 2026). Building on these findings, the present study further expands the biological application of CV122 by identifying HPSE-mediated glycocalyx remodeling as a key mechanism underlying airway epithelial barrier dysfunction during COPD progression (Figure 5). In the present study, we demonstrate that HPSE-associated glycocalyx remodeling is an important contributor to airway epithelial barrier dysfunction in COPD. We further show that the novel small-molecule inhibitor CV122, a previously reported compound developed by our group, exerts significant protective effects through pharmacological inhibition of HPSE, both in experimental animal models and *in vitro* epithelial systems. Although our previous work established the biochemical inhibitory activity of CV122 against HPSE, the present study extends these findings by identifying airway epithelial glycocalyx remodeling and barrier dysfunction as disease-relevant downstream processes associated with HPSE inhibition in COPD.

**FIGURE 5:**
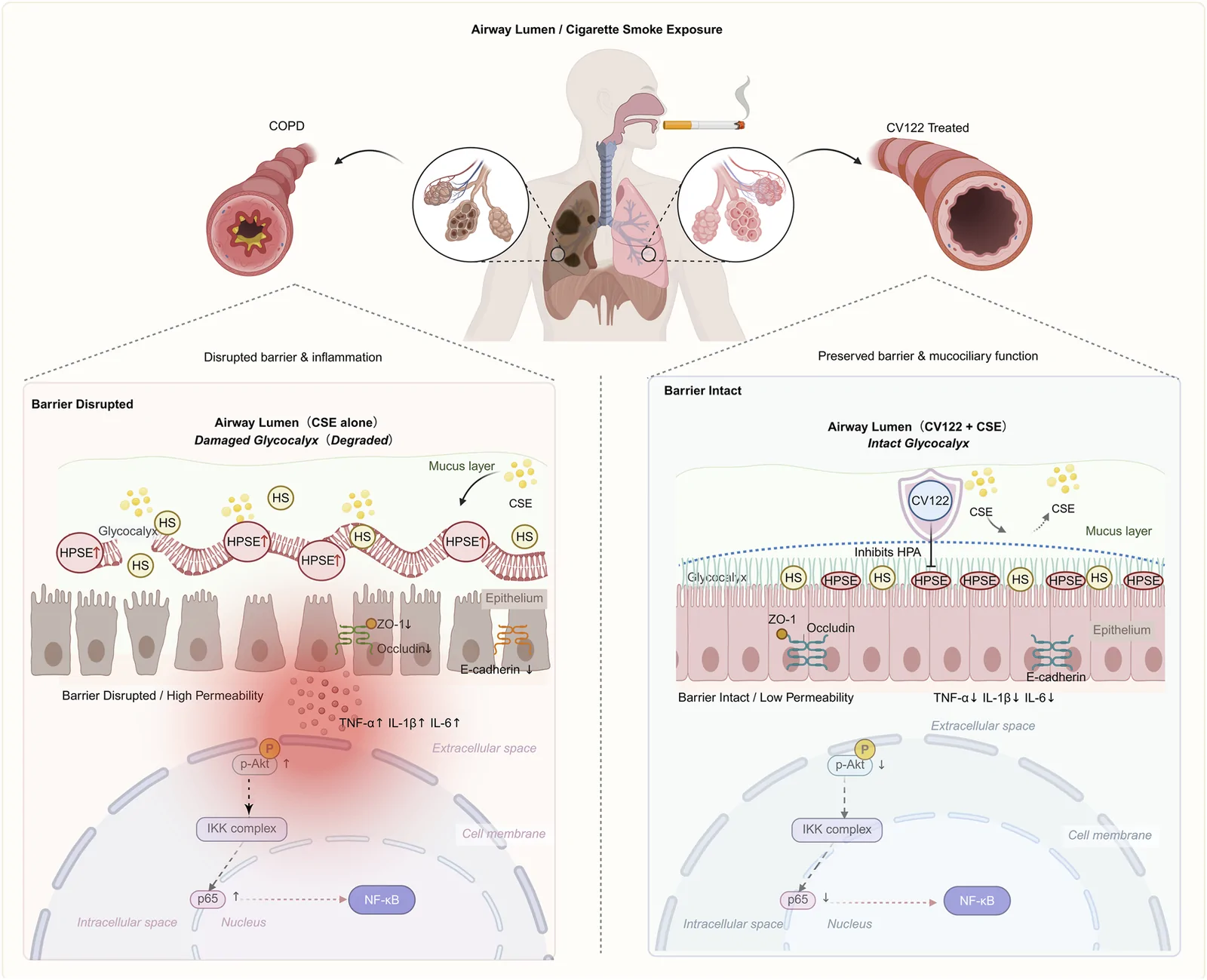
CV122 alleviates cigarette smoke–induced airway epithelial injury by targeting the HPSE–glycocalyx–Akt/NF-κB axis.

Our findings elucidate how environmental stimuli, such as cigarette smoke, contribute to structural barrier disruption and persistent inflammation, while highlighting the glycocalyx-barrier axis as a previously underappreciated therapeutic target in COPD. Airway epithelial barrier dysfunction is increasingly recognized as a central event in COPD pathogenesis, linking environmental exposures to chronic inflammation and tissue remodeling (Ji et al., 2026; Hewitt and Lloyd, 2021). Although extensive studies have documented the disruption of tight junctions, adherens junctions, and the glycocalyx layer, the upstream mechanisms initiating barrier breakdown remain incompletely understood (Li et al., 2021; Zihni et al., 2016; Raby et al., 2023; Guo et al., 2025). Here, we show that HPSE expression is consistently elevated in COPD patients, smoke-exposed mice, and CSE-treated epithelial cells, suggesting that HPSE upregulation represents a conserved response to injurious stimuli. It should be noted that assessment of HPSE protein abundance remains biologically meaningful despite not directly reflecting enzymatic activity. HPSE is the major mammalian endoglycosidase responsible for HS cleavage, and increased HPSE expression represents an essential prerequisite for subsequent proteolytic processing and catalytic function. Moreover, sustained HPSE overexpression has been implicated in glycocalyx remodeling and amplification of inflammatory responses in multiple pathological conditions. Therefore, increased HPSE abundance reflects enhanced involvement of the HPSE–HS remodeling axis during disease progression, although it should not be interpreted as direct evidence of increased enzymatic activity. Notably, the dynamic pattern of HPSE expression observed *in vivo* indicates that HPSE may play a particularly critical role during the early stages of epithelial injury, potentially acting as a trigger for subsequent pathological cascades. Mechanistically, we demonstrate that HPSE-mediated degradation of HS disrupts the structural integrity of the epithelial glycocalyx, thereby impairing barrier function at multiple levels. The glycocalyx serves not only as a physical barrier but also as a regulator of cytokine gradients and cellular signaling. Its degradation leads to increased epithelial permeability, release of bound inflammatory mediators, and amplification of local inflammatory responses. Importantly, we observed that glycocalyx disruption is closely associated with the downregulation of key junctional proteins, including ZO-1, occludin, and E-cadherin, indicating coordinated impairment of both biochemical and structural components of the epithelial barrier. It should be noted that WGA staining reflects overall glycocalyx-associated carbohydrate structures rather than HS specifically. Therefore, WGA staining alone cannot be interpreted as direct evidence of HPSE-mediated HS degradation. To address this limitation, the present study further incorporated HS-specific immunofluorescence together with ELISA-based quantification of HS in serum and cell culture supernatants. These complementary analyses provide direct evidence of HS remodeling and support an association between increased HPSE abundance and glycocalyx disruption.

These findings provide evidence for a functional association between glycocalyx integrity and junctional stability, filling an important gap in understanding epithelial barrier regulation in COPD. Beyond its association with structural glycocalyx remodeling, HPSE upregulation and increased abundance of the mature active form may also be linked to inflammatory signaling. Our results show that cigarette smoke exposure activates the Akt/NF-κB pathway, consistent with previous studies implicating this pathway in inflammation (Li et al., 2024; Yang et al., 2022; Wu et al., 2025). Notably, treatment with CV122 effectively suppressed Akt and NF-κB phosphorylation, indicating an association between pharmacological HPSE inhibition and attenuation of Akt/NF-κB signaling. This observation is consistent with previous studies suggesting that glycocalyx degradation may facilitate the release and diffusion of pro-inflammatory mediators, thereby amplifying intracellular signaling responses. Collectively, these findings suggest that HPSE may participate in coordinating structural glycocalyx remodeling and inflammatory signaling in airway epithelial cells. From a translational perspective, our findings support HPSE inhibition as a potential strategy for attenuating COPD-associated epithelial injury. The broad protective effects of CV122 across glycocalyx integrity, epithelial barrier function, inflammatory responses, and tissue remodeling suggest that HPSE may occupy a regulatory position linking structural epithelial injury with inflammatory amplification. However, these effects may also reflect interconnected downstream consequences of reduced tissue injury rather than independent direct actions of HPSE inhibition on each pathological process. Together, these findings indicate that targeting HPSE may simultaneously modulate multiple pathological processes, including barrier dysfunction, inflammation, and tissue remodeling. Although these findings support HPSE inhibition as a promising therapeutic strategy, it should be noted that CV122 administration in the present study was initiated simultaneously with cigarette smoke/LPS exposure. Therefore, the current experimental design primarily evaluates the preventive or disease-attenuating effects of HPSE inhibition during COPD development rather than therapeutic efficacy in established disease. Future studies employing delayed intervention after establishment of COPD will be necessary to determine the therapeutic potential of CV122 under clinically relevant conditions.

Despite these strengths, several limitations should be acknowledged. First, although our data reveal a strong association between glycocalyx degradation and junctional protein disruption, the precise molecular mechanisms linking these processes require further investigation. Second, although the processed active 50-kDa HPSE form was quantified in the present study, direct biochemical measurement of endogenous HPSE enzymatic activity was not performed. Therefore, the precise contribution of catalytic HPSE activation, as well as its regulatory relationship with downstream Akt/NF-κB signaling during COPD progression, remains to be further elucidated. Future studies incorporating dedicated enzymatic activity assays will further clarify the contribution of catalytic HPSE activation during COPD progression. Third, while CV122 shows promising efficacy in preclinical models, its pharmacokinetics, safety profile, and long-term effects remain to be evaluated in clinical settings. Fourth, the heterogeneity of COPD, including variations in disease stage and phenotype, may influence the therapeutic response to HPSE inhibition and warrants further study. Finally, the human validation cohort was relatively small and lacked detailed clinical information because of the retrospective nature of tissue collection. Therefore, the human findings should be considered supportive preliminary validation and require confirmation in larger, well-characterized patient cohorts. Future studies employing more physiologically relevant human airway epithelial systems, including primary airway epithelial cells cultured at an air–liquid interface and patient-derived airway organoids, will be important to further validate the present findings and evaluate the translational potential of HPSE inhibition in COPD.

In summary, the present study supports an important role of HPSE in airway epithelial barrier dysfunction during COPD progression and identifies glycocalyx remodeling as a key pathological event associated with disease development. Pharmacological inhibition of HPSE with CV122 preserves epithelial barrier function and attenuates COPD-like pathological changes in experimental models, supporting HPSE as a promising therapeutic target that warrants further investigation.

## 5 Declarations

The authors declare that they have no known competing financial interests or personal relationships that could have appeared to influence the work reported in this paper.

## Data Availability

The original contributions presented in the study are included in the article/Supplementary Material, further inquiries can be directed to the corresponding authors.
