# Supplementary material for: Targeting heparanase-associated epithelial glycocalyx remodeling attenuates Akt/NF-κB–related barrier dysfunction in experimental COPD
Source: Front Pharmacol. 2026 Sep 21;17:1934045. doi: 10.3389/fphar.2026.1934045 (PMC13634845; doi:10.3389/fphar.2026.1934045)
Supplement: Supplementary file 1 [file Supplementaryfile1.docx]

***Supporting Information***

**Targeting Heparanase-Dependent Epithelial Glycocalyx Remodeling Attenuates Akt/NF-κB–Mediated Barrier Dysfunction in Experimental COPD**

**Yueping Bai^1,2^; Xushan Ma^1^; Hongzhen Jin^2^; Junna Song^1^; Xiaoxuan Zeng^1^; Fengjuan Liu^1,2^; He Fang^1,2^; Xiao Wu^2,^*; Song Yang^2,^*; Wei Zhao^1,2,^***

^1^*State Key Laboratory of Medicinal Chemical Biology, College of Pharmacy, Key Laboratory of Molecular Drug Research and KLMDASR of Tianjin, Nankai University, Tianjin 300350, China*

^2^*Qingdao Central Hospital, NHC Key Laboratory of Cardiopulmonary Rehabilitation and Functional Recovery, Industry-Academia-Research Collaborative and Innovation Center for Intelligent Rehabilitation Drug R&D of Shandong Province, Qingdao Key Laboratory of Precision Drug Research for Chronic Disease Rehabilitation, School of Health and Life Sciences, University of Health and Rehabilitation Sciences, Qingdao 266113, China*

*****Corresponding authors: E-mail addresses: [wuxiao@uhrs.edu.cn](mailto:wuxiao@uhrs.edu.cn) (Xiao Wu), yangsong@uhrs.edu.cn (Song Yang), wzhao@nankai.edu.cn (Wei Zhao)

1. **Supplementary Figures**

**Figure S1. Progressive structural remodeling and HPSE upregulation in COPD development and CSE-induced epithelial injury.** (A) Representative H&E staining of lung tissues from control mice and COPD mice at 15, 30, 45, and 60 days, with quantitative analysis of MLI indicating progressive alveolar destruction. (B) Representative Masson’s trichrome staining and quantification of collagen deposition (fibrotic area %) in lung tissues during COPD progression. (C) Quantification of HPSE-positive cells in lung tissues from control and COPD mice. (D) Quantification of HPSE fluorescence intensity in lung tissues. (E) Cell viability of BEAS-2B cells exposed to increasing concentrations of CSE for 12 h, assessed by MTT assay. (F) Quantification of HPSE fluorescence intensity in BEAS-2B cells following CSE stimulation. Data are presented as mean ± SD. Mouse experiments: n = 5 mice per group. *P < 0.05, **P < 0.01, ***P < 0.001, ****P < 0.0001; ns, not significant.

**Figure S2. Quantitative analysis of inflammatory cell populations in BALF from COPD mice following CV122 treatment.** (A) Macrophage counts. (B) Neutrophil counts. (C) Lymphocyte counts. Data are presented as mean ± SD. Mouse experiments: n = 5 mice per group. *P < 0.05, **P < 0.01, ***P < 0.001, ****P < 0.0001; ns, not significant.

**Figure S3.** Healthy mice received **CV122** (20 mg/kg) for the same duration as the COPD treatment protocol. Long-term **CV122** administration does not alter pulmonary physiology or glycocalyx integrity in healthy mice. (A-C) Pulmonary function analysis, including (FVC), FEV0.3, and Cdyn. (D) Representative H&E staining of lung tissues and corresponding high-magnification images showing normal pulmonary architecture following **CV122** administration. (E) Quantification of MLI. (F) Representative WGA staining of pulmonary glycocalyx in control and **CV122** -treated mice. (G) Quantification of WGA fluorescence intensity. Data are presented as mean ± SD. Mouse experiments: n = 5 mice per group. ns, not significant.

**Figure S4. CV122 reduces HPSE expression in lung tissues of COPD mice.** (A) Representative immunohistochemical staining of HPSE in lung tissues from control, COPD, and CV122-treated mice. (B) Quantification of HPSE-positive area (%) in lung tissues. (C) HPSE levels in BALF measured by ELISA. Data are presented as mean ± SD. *P < 0.05, **P < 0.01, ***P < 0.001, ****P < 0.0001; ns, not significant.

**Figure S5. Quantification of epithelial junction protein immunofluorescence in lung tissues from COPD mice following CV122 treatment.** (A) E-cadherin fluorescence intensity. (B) ZO-1 fluorescence intensity. (C) Occludin fluorescence intensity. Data are presented as mean ± SD. Mouse experiments: n = 5 mice per group. *P < 0.05, **P < 0.01, ***P < 0.001, ****P < 0.0001; ns, not significant.

2. **Supplementary Tables**

**Supplementary Table 1.** Demographic data for control subjects and patients with COPD

| Study ID | Age | Sex | Race |
| --- | --- | --- | --- |
| COPD1 | 62 | Male | Asian individuals |
| COPD2 | 70 | Male |  |
| COPD3 | 69 | Male |  |
| COPD4 | 80 | Male |  |
| COPD5 | 70 | Male |  |
| COPD6 | 68 | Male |  |
| COPD7 | 71 | Female |  |
| Control1 | 62 | Female |  |
| Control2 | 58 | Male |  |
| Control3 | 68 | Male |  |
| Control4 | 55 | Male |  |
